# Global Needs and Barriers for Medical Research Education: Initiatives to Solve the Physician-Scientist Shortage

**DOI:** 10.5195/ijms.2023.2011

**Published:** 2023-03-31

**Authors:** Marc R. Schneider, Abdelrahman M. Makram, Esther Bassey, Mihnea-Alexandru Găman, Ciara Egan, Juan C. Puyana, Francisco J. Bonilla-Escobar

**Affiliations:** 1 Faculty of Medicine, Medical University of Varna, 9000 Varna, Bulgaria, Student Editor, IJMS.; 2 School of Public Health, Imperial College London, London, United Kingdom, Student Editor, IJMS.; 3 Faculty of Basic Medical Sciences, University of Uyo, Nigeria, Student Editor, IJMS.; 4 Faculty of Medicine, “Carol Davila” University of Medicine and Pharmacy, 050474 Bucharest, Romania & Department of Hematology, Center of Hematology and Bone Marrow Transplantation, Fundeni Clinical Institute, 022328 Bucharest, Romania. Scientific Editor, IJMS; 5 Humanitas University, Humanitas Research Hospital, Milan, Italy. Deputy Editor, IJMS.; 6 School of Medicine, Department of Surgery, Professor of Surgery, Critical Care Medicine, and Clinical Translational Science, Director for Global Health-Surgery, University of Pittsburgh, Pittsburgh, PA, United States. Editorial Board Member, IJMS.; 7 Department of Ophtalmology; Institute for Clinical Research Education (ICRE), University of Pittsburgh, Pittsburgh, PA, United States. Fundación Somos Ciencia al Servicio de la Comunidad, Fundación SCISCO/Science to Serve the Community Foundation, SCISCO Foundation, Cali Colombia. Grupo de investigación en Visión y Salud Ocular, VISOC, Universidad del Valle, Cali, Colombia. Editor in Chief, IJMS.

In a recent interview, Dr. Anthony Fauci said the words that physicians worldwide must embrace and apply: “…You’re a scientist.”^1^ There is a little romanticism in the belief that all physicians are scientists, but it is our deepest wish that all physicians find a passion for research. It is easier said than done. Research requires rigor, knowledge, the use of validated methods, resources, mentors, adherence to ethical guidelines, sometimes the endurance and hardships of data collection, the ability to write in a “scientific manner”, and the patience to get the results published. Through all those barriers is where we, as a society, begin to lose physician-scientists from the tracks. People like research until they find the above-mentioned barriers or get tired of fighting against them, and this can happen at any stage of a physician’s career, as a medical student, resident, clinician, or faculty.

Moving away from barriers after medical school, medical students’ education includes the encouragement of independent study to stay up to date. Some efforts are also made to train them as potentially independent researchers. These efforts sometimes are just shy attempts to teach research methodologies and statistics without proper applicability or the resources to carry out real-life research. One of the very few ways to get real training in most of the cases is to pursue a graduate diploma in research being a master’s or a Ph.D. or a combined program (i.e., MD/MSc, MD/Ph.D., etc.). Nevertheless, and besides all the barriers that a medical student can face to carry out research, some (should be everyone but that will be discussed in the next editorial) reach the goal of a scientific publication. A scientist not only reads critically, but they must also write and publish. We at the International Journal of Medical Students are committed to the publication of medical students and early career-scientist research and we try our best to help authors get their papers out. Our innovative, 10-year tested and validated, two-step peer-reviewing process help us achieve high quality early-career scientists publications.^2–4^ It includes a step of real peers (medical students with publications) reviewing and suggesting ways to improve the research or the way that is being described or analyzed to make it into a publishable scientific article.

Medical student barriers to research have been widely studied and still, the issue has not been fully addressed. Several different efforts, like journals focusing on medical students’ research or initiatives to mentor and help medical students to carry out research (https://ijms.info/IJMS/Conference/sponsors) are among the few options that medical students have to become those scientists that the world needs.^5–7^ Humankind is lucky enough that some medical students are not satisfied with what medical school provides. They go the extra mile to change their environment to be able to become scientists. In this issue, we found some interesting research and medical students’ activities that are aligned with the above arguments. Medical students experience more barriers to research compared to residents or faculty, and still they are asked to have publications or research experience to get into a residency. This can go against the promotion of research and could encourage predatory publishing behaviors.^9^ These barriers to do research are reinforced by gender bias and its effects on women wanting to pursue a career in science.^10^ Mentorship and medical curricula reforms are needed to encourage medical students to explore the field of medical research,^9^ and evidence-based decisions,^11,12^ ensuring that they will continue publishing after graduating,^13,14^ have better employment opportunities,^13^ and can be better clinicians.^15^

## How to Make Editors and Scientists: An IJMS Initiative

For a decade, the IJMS has striven to develop an international society of young scientists via education and mentorship. Building opportunities for the community of medical students and freshly graduated physicians has led to immense growth not just for the Journal itself and medical students’ research quality, but also for the Editorial Team behind the journal.

The nourishing soil for this successful history is founded on integrity and the will to develop a new generation of editors and scientists.^16^ Training via the Web of Science Academy and the Committee on Publication Ethics (COPE) courses constitutes the backbone of the Journal and is mandatory for the whole Editorial Team, which also underlines the objective of the IJMS: to be the leading platform for early-career scientists’ medical research. The generations of physician-scientists that have been part of IJMS are highlighted on our Alumni website section: https://ijms.info/IJMS/about/editorialTeam/alumni

The IJMS creates opportunities for medical students to get involved in the development of original articles. The initiative strives to bring young scientists together and mentor them to improve their research. Having built a network of more than sixty young research enthusiasts, we are happy to share that there are numerous research collaborations within the IJMS network that are of the highest quality and published in high-impact journals. We see the successfully published articles of these collaborations as a transcript of our efforts. The manuscripts tackled topics in the fields of hematology and oncology,^17–23^ ethics (e.g., diversity, equity and inclusion),^24–27^ infectious diseases,^28,29^ surgery,^30,31^ pediatrics,^32^ and physical and rehabilitation medicine.^33–35^ We invite you to read them and encourage you to include students and early career scientists in your research projects.

The quality of our team is also expressed in the statistics of the Journal. In 2022, excluding editorials, our team was able to process a total of 58 articles from submission to publication (acceptance rate=29%). We were able to provide the first decision in only four days for most papers. The authors received the first revision request on an average of 49.2 days (SD=63.4, median=32.5, IQR: 23–49, range: 3–433) after the initial submission. With this fast pace, we were also able to accept articles on average after 187.9 days (SD=130.1, median=147.5, IQR: 114–224, range: 34–662) from the initial submission. And finally, from acceptance to publication, it took an average of 31.8 days (SD=40.1, median=9.5, IQR: 3–53, range: 0–145) to produce publishable articles.

## Announcement of the 2023 World Conference of Medical Student Research

After steadily gaining popularity and a successful conference in 2022, the IJMS is pleased to announce the 2023 World Conference of Medical Student Research (WCMSR) to be held on October 7th, 2023. Sparking the idea to foster a new generation of physician-scientists and to enable international networking, we aim to further develop the WCMSR as the fundament for an inclusive scientific society. Again, the format will be fully online allowing participants to present their research to an international audience.^36^ The presentations will be evaluated by an expert jury, and the top 10 works will have the possibility of a fast-track peer-reviewing process. Each abstract accepted will receive its own digital object identifier (DOI) and publication in Volume 11 Supplement 1 in December 2023. Please refer to the website for more information: https://ijms.info/IJMS/Conference/welcome.

## Other Papers in This Issue

Turning away from the training aspect, this issue features an interesting study by Zhou et al. The authors propose the use of a new decell process in tracheal transplantation that involves removal of epithelial, mucosal, and submucosal cells while preserving chondrocytes. This procedure was carried out on the trachea of Yorkshire pigs and achieved by an adaptation of the sodium dodecyl sulfate cycle protocol. This novel procedure particularly improves the short-term viability of chondrocytes with the limitation on long-term viability.^37^

Soleymani et al.’s manuscript focuses on promoting physical activity amongst people who use wheelchairs by predicting the accuracy and precision of research tools, e.g., ActiGraph accelerometers and SMARTwheels, which can be used to measure pushes. The study reports that SMARTwheels had a minor undercount of the total number of pushes across workloads versus ActiGraph accelerometers (worn on the arm) which recorded an overcount of the same parameters. Thus, the authors advocate for more accurate research in this area.^38^

Ng and Velanthren report the case of a 69-year-old Chinese man in Malaysia with a windswept deformity caused by pseudogout. The patient presented with pain in both knees and right shoulder, and deformed windswept knee requiring the use of a walking stick, with full painless flexion in both knees and shoulders. Imaging studies showed complete destruction of the knee joints. As the patient could not afford surgery, he was placed on steroids and colchicine. This case report shows how devastating pseudogout can be and the need for affordable surgical care to promote health equity.^39^

Munoz-Valencia et al. described an association between the availability of blood banks and the incidence of deaths due to traumatic hemorrhagic shock. The authors used national datasets from Colombia and analyzed clusters of municipalities. They concluded that the more banks the lower the incidence of deaths due hemorrhagic shock. Interestingly, the authors mention the lack of research on geographic determinants of blood products’ availability and the need for granular data to enhance conclusions.^40^

Peñafiel-Pallares et al. report the case of a 51-year-old female presenting with a three-month history of headache and diplopia. She was found with fundoscopic abnormalities, with absent clinical history of hemophilia. She was diagnosed with an uncommon combination of a hemophilic protein C type 1 deficiency and a cavernous sinus thrombosis complicating sphenoidal rhinosinusitis. The authors point out the importance of early recognition and treatment in such cases.^41^

Revisiting the beginning of a worldwide catastrophe, Gajare et al. share their experiences during their COVID-19 travelers screening activity by explaining protocols, guidelines, and screening procedures of international passengers for symptoms of COVID-19 at one of the largest airports in India.^42^

As suicide is the second leading cause of death worldwide in 15-29-year-olds, Keuch et al. investigate the prevalence of suicidal ideation as an indicator for future suicide attempts among German medical students and suggest further development and implementation of preventive strategies.^43^

Gabralla et al. present a case of exacerbated, previously well-controlled, myasthenia gravis (MG) following the second dose of the AstraZeneca COVID-19 vaccine. Despite rehydration therapy and steroids, the 37-year-old patient passed away after failed attempts to obtain IV immunoglobulins.^44^ Although this is not the first MG case to suffer from disease exacerbation after receiving the COVID-19 vaccination,^45–49^ nearly a fifth of MG patients were found to experience exacerbations during or after COVID-19 infection in a study conducted by Peric et al.^50^

Even if high-income countries have limited the wearing of white coats in healthcare facilities to decrease hospital-acquired infections or healthcare-associated infections (HAIs),^51^ most of the world still sticks to wearing them. Daraniyagala et al. conducted a cross-sectional study on medical students to assess the rate of white coat contamination, the responsible pathogens, and antibiotic resistance phenotypes. Nearly half of the participants had coats contaminated with bacteria associated with HAIs, including methicillin-resistant Staphylococcus aureus or vancomycin-resistant enterococci. Therefore, it is important to limit wearing white coats and switch to scrubs, or to have strict rules as to how and when to wear white coats.^52^

## Conclusion

In a world where changes are inevitable and challenges particularly in healthcare emerge every day, research has become an essential problem-solving tool. Physician-scientists are needed more than ever. The IJMS has and will continue to provide an open platform for medical students and junior doctors to explore their areas of interest in research and share their findings with their peers and the world at large. Our platform has also encouraged collaborations across the world and led to the publication of innovative research papers. Furthermore, the introduction of the WCMSR is another mind-blowing progress to unveiling the work done amongst the students and junior doctors. The IJMS is on a wheel of progression, and in the near future, an even bigger impact will be made to achieve the goal of creating positive change in healthcare through research in the global society.
